# Multiparametric Characterization of Intracranial Gliomas Using Dynamic [18F]FET-PET and Magnetic Resonance Spectroscopy

**DOI:** 10.3390/diagnostics12102331

**Published:** 2022-09-27

**Authors:** Thomas Pyka, Iwona Krzyzanowska, Axel Rominger, Claire Delbridge, Bernhard Meyer, Tobias Boeckh-Behrens, Claus Zimmer, Jens Gempt

**Affiliations:** 1 Department of Nuclear Medicine, Klinikum Rechts der Isar, Technische Universität München, Ismaninger Str. 22, D-81675 München, Germany; 2 Department of Neuroradiology, Klinikum Rechts der Isar, Technische Universität München, Ismaninger Str. 22, D-81675 München, Germany; 3 Department of Nuclear Medicine, Inselspital, Bern University Hospital, Freiburgstr. 18, CH-3013 Bern, Switzerland; 4 Department of Neurosurgery, Klinikum Rechts der Isar, Technische Universität München, Ismaninger Str. 22, D-81675 München, Germany; 5 Institute of Neuropathology, Institute of Pathology, Klinikum Rechts der Isar, Technische Universität München, Ismaninger Str. 22, D-81675 München, Germany

**Keywords:** magnetic resonance imaging, positron emission tomography, glioma, magnetic resonance spectroscopy, multiparametric imaging

## Abstract

Both static and dynamic *O*-(2-[18F]fluoroethyl)-l-tyrosine-(FET)-PET and 1H magnetic resonance spectroscopy (MRS) are useful tools for grading and prognostication in gliomas. However, little is known about the potential of multimodal imaging comprising both procedures. We therefore acquired NAA/Cr and Cho/Cr ratios in multi-voxel MRS as well as FET-PET parameters in 67 glioma patients and determined multiparametric parameter combinations. Using receiver operating characteristics, differentiation between low-grade and high-grade glioma was possible by static FET-PET (area under the curve (AUC) 0.86, *p* = 0.001), time-to-peak (TTP; AUC 0.79, *p* = 0.049), and using the Cho/Cr ratio (AUC 0.72, *p* = 0.039), while the multimodal analysis led to improved discrimination with an AUC of 0.97 (*p* = 0.001). In order to distinguish glioblastoma from non-glioblastoma, MRS (NAA/Cr ratio, AUC 0.66, *p* = 0.031), and dynamic FET-PET (AUC 0.88, *p* = 0.001) were superior to static FET imaging. The multimodal analysis increased the accuracy with an AUC of 0.97 (*p* < 0.001). In the survival analysis, PET parameters, but not spectroscopy, were significantly correlated with overall survival (OS, static PET *p* = 0.014, TTP *p* = 0.012), still, the multiparametric analysis, including MRS, was also useful for the prediction of OS (*p* = 0.002). In conclusion, FET-PET and MRS provide complementary information to better characterize gliomas before therapy, which is particularly interesting with respect to the increasing use of hybrid PET/MRI for brain tumors.

## 1. Introduction

A conventional magnetic resonance imaging (MRI) is a mainstay in glioma imaging, but it has been considered insufficient to approach questions, such as tumor grading, delineation of tumor margins, and differentiation between viable tumor and post-therapeutic changes. Supplementing information can be provided by molecular imaging, e.g., using 1H magnetic resonance spectroscopy (MRS) or positron emission tomography (PET) with amino acid tracers. MRS aims at determining the concentration of specific metabolites and their ratios, the most important ones being N-acetyl-aspartate (NAA), choline (Ch), and creatine (Cr) [1]. Amino acid PET, on the other hand, depicts changes in amino acid uptake in different types of lesions [2]. For the latter, *O*-(2-[18F]fluoroethyl)-l-tyrosine (FET) is used increasingly over [11C]methionine, due to better availability and easier distribution. Several authors have already shown the advantage of MRS [3,4,5] and FET-PET [6,7,8,9,10,11,12] compared to morphologic MRI alone, which has led to the development of joint guidelines for the inclusion of FET-PET imaging in the work-up of gliomas [13]. Moreover, the advent of integrated PET/MRI offers the possibility of combining MRS and PET in a single imaging session [14,15]. Only a few studies, however, have investigated the correlation between the two modalities and their possible added value, which would be of high interest when establishing hybrid imaging protocols [16]. Recently, an interesting study compared the value of FET-PET, conventional, and advanced MR imaging for the detection of tumor infiltration in low and high-grade gliomas, showing added value in high-grade tumors for combining FET-PET with apparent diffusion coefficient (ADC) maps, but not MRS [17].

Herein, we focus on the multimodal assessment of gliomas with respect to grading and prognostication. Dunet et al. [18] published results on the differentiation between low- and high-grade gliomas with FET-PET, MRS, and diffusion-weighted imaging, but included only 38 patients and did not provide an analysis of the patient outcomes. In addition, only a single-voxel MRS and no N-acetyl-aspartate (NAA) ratios were employed; perhaps as a consequence, the study did not yield significant results for MRS, which is in contrast to recent meta-analyses [5,19]. In our study, we investigated a larger cohort of patients receiving FET-PET, with a proportion of patients undergoing dynamic PET imaging, and multi-voxel MRS; we attempted to validate earlier results on the diagnostic value of the modalities and establish the benefits provided by multimodal imaging.

## 2. Materials and Methods

### 2.1. Data Collection

From our database, we retrospectively identified 67 consecutive patients with intracranial gliomas (60 first diagnosis, 7 recurrent) who had received multivoxel MRS and FET-PET (delay between the imaging procedures 5.6 +/− 8.6 days). In all patients, either surgical resection or biopsy was performed immediately after the imaging procedure (6.7 +/− 6.3 days after MRS). Histological world health organization (WHO grade was determined by a board-certified and experienced neuropathologist according to the WHO Classification of Tumors of the Central Nervous System 4th Edition [20]. For the recurrent tumors, any significant radiation necrosis potentially altering the results could be excluded. Static FET-PET and MRS were available for all patients, while dynamic PET scans were obtained in 25 cases (23 first diagnosed, 2 recurrent). Overall survival from the date of MRS was assessed, excluding patients with recurrent tumors.

### 2.2. Proton MR Spectroscopic Imaging

Multi voxel MRS was performed on a 3 Tesla MR scanner (Achieva, Philips N.V., Eindhoven, The Netherlands) as previously described [21], with technical specifications as follows: PRESS sequence, TR: 1669 ms, TE: 288 ms, FOV 200 (ap) × 176 (rl) × 72 (fh) mm, 10 mm isovoxel. Planning the spectroscopic scan volume was done on a post-contrast T1w 3D MPRAGE (TR: 9 ms, TE: 4 ms, FOV 240 (ap) × 240 (rl) × 160 (fh) mm, voxel size: 1 mm^3^, acquisition time: 5:56 min). Postprocessing included water subtraction, apodization filtering, zero filling, and fusion with the anatomical, and was performed with the software “SpectroView” on an extended MR workspace workstation (Philips N.V.). Maximum NAA/Cr and Cho/Cr ratios were determined in tumor tissue. To achieve this, up to twenty voxels per patient with the highest quality were selected under the supervision of a board-certified neuroradiologist with several years of experience in spectroscopic imaging. For comparison, values were also calculated in the contralateral hemisphere. Using these highest quality voxels, the maximum values for the metabolite ratios were extracted.

### 2.3. FET-PET Imaging

PET scans were obtained with a ECAT EXACT HR+ scanner (Siemens AG, Erlangen, Germany) at the University Hospital of the Technical University of Munich. To achieve standardized metabolic conditions, patients had to fast for a minimum of 6 h before undergoing scanning. After a transmission scan with 68-Ge sources (duration 15 min), a target dose of 190 MBq of FET was injected. Dynamic studies were acquired up to 40 min after injection (128 × 128 matrix, 3-dimensional mode), comprising 16 timeframes (7 × 10 s, 3 × 30 s, 2 min, 3 × 5 min, and 2 × 10 min). Data were reconstructed by filtered back projection using a Hann filter with a cut-off frequency of 0.34 Nyquist and corrected for scattering and attenuation. For static data, mean tumor-to-background ratios (TBRmean) were calculated using a 30–40 min summed frame with a circular 1-cm region of interest (ROI)ac around the spot of highest tracer uptake and a contralateral background ROI according to standard criteria. Dynamic time–activity curves were determined in a 90% isocontour ROI around the hottest voxel as described previously [11]. Based on the time–activity curves, the time to peak (TTP) of the time activity curve was extracted.

### 2.4. Statistical Analysis

For each patient, we tested the ability of the Cho/Cr and NAA/Cr ratios as well as TBRmean and dynamic FET TTP to differentiate between WHO grades using receiver operating characteristic (ROC) analysis. The area under the ROC curve (AUC) and optimal discrimination threshold, as well as the sensitivity and specificity, were calculated. Correlation between MRS and PET parameters and overall survival (OS) was performed using Kaplan–Meier analysis (log-rank test) and multivariate Cox regression. Optimal thresholds for distinguishing low-risk and high-risk groups were determined by the online application Cutoff Finder (https://molpathoheidelberg.shinyapps.io/CutoffFinder_v1/, accessed on 27 July 2022 [22]). SPSS 24 (IBM, Armonk, NY, USA) was used for all other statistics. For multimodal analysis, we determined an optimal combination of the MRS and PET parameters by logistic regression or Cox regression, respectively. *p*-values of 0.05 or less were considered statistically significant.

## 3. Results

**Figure 1:**
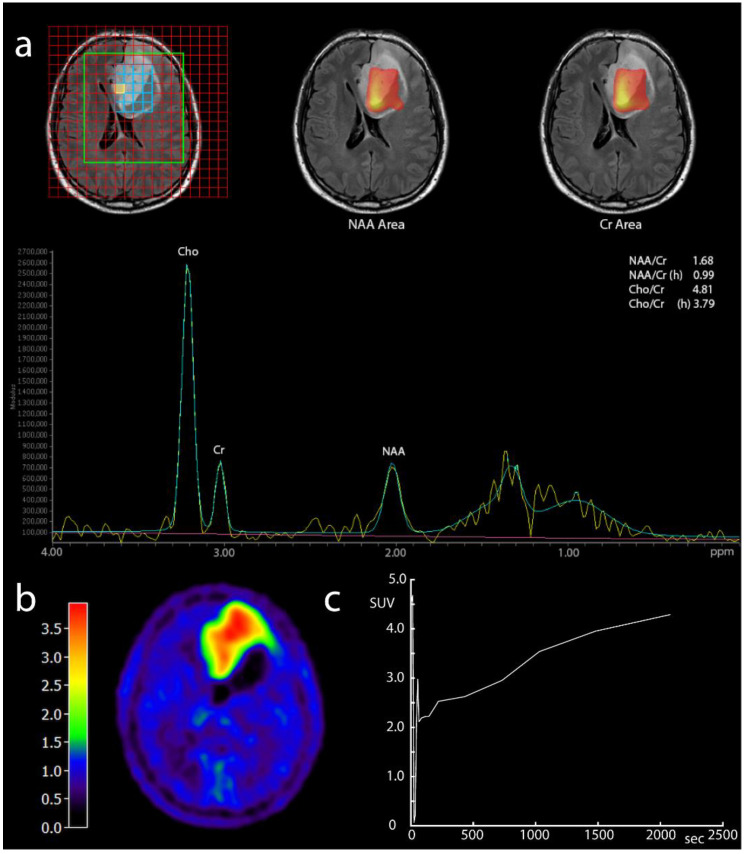
Example MR spectroscopy and FET-PET in a patient with glioblastoma; 53-year-old patient with untreated left frontal glioblastoma (WHO grade IV). (**a**) Multi-voxel 1H-MRS with spectroscopy grid and representative spectrum from a tumor voxel with signal intensities and ratios for Cho, Cr, and NAA, showing an elevated Cho/Cr ratio. Yellow line—unsmoothed, green line—smoothed values, purple - baseline. (**b**) Static FET-PET scan 30 min after injection with intense tracer enhancement (TBR 3.5). (**c**) Dynamic FET-PET over 40 min shows a linearly ascending activity slope in the tumor, untypical for glioblastoma. Interestingly, this patient had an unusual benign course with one relapse but long-term survival over several years and no further recurrence so far.

Out of 67 patients, 9 (13.5%) had WHO grade II gliomas, 14 (20.9%) had WHO grade III gliomas, and 44 (65.7%) patients had glioblastomas. At the time of our analysis, out of 60 patients with the initial diagnosis of glioma at the time of imaging, 23 patients (38.3%) were alive, and the median OS was 12.4 months (range 0.7–78.8 months). The demographic and clinical characteristics of all study patients are summarized in Table 1. An example of multiparametric imaging in a glioblastoma patient is depicted in Figure 1.

### 3.1. Differentiation between Low-Grade and High-Grade Gliomas

**Figure 2:**
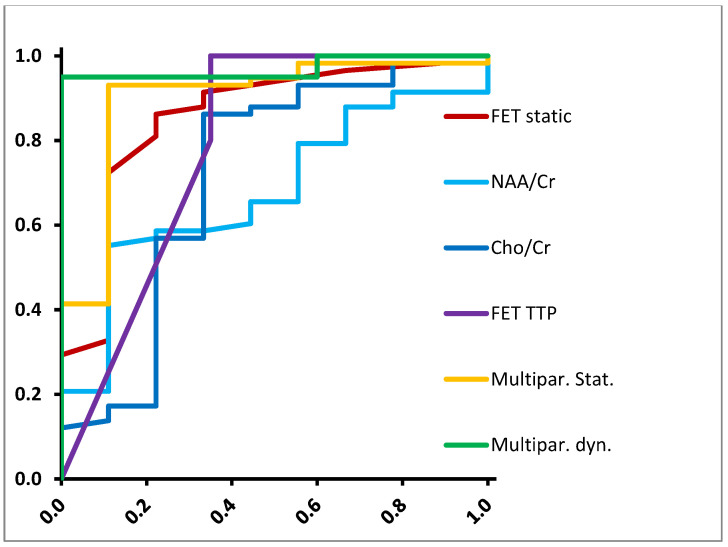
ROC curves for differentiation between low-grade and high-grade gliomas.

First, we tested the ability of different parameters to distinguish between low-grade (up to WHO °II) and high-grade (WHO °III–IV) gliomas (Table 2, Figure 2). Static FET-PET was the best single parameter showing 86% sensitivity and 78% specificity (AUC 0.855, *p* = 0.001), with an optimal threshold of TBR 2.00. MRS yielded sensitivities and specificities of 86% and 67% for Cho/Cr ratios (AUC 0.716, *p* = 0.039, optimal threshold: 2.22) and 66% and 56% for NAA/Cr ratios (AUC 0.678, *p* = 0.089, threshold: 1.89). Dynamic FET-PET TTP was also able to discriminate between low-grade and high-grade on a significant level (AUC 0.790, *p* = 0.049). With a multiparametric approach using static FET uptake and NAA/Cr ratio, the diagnostic accuracy could be improved to 93% sensitivity and 89% specificity (AUC 0.898, *p* < 0.001). The coefficients (β) for the logistic model were calculated as 2.533 for TBRmean and 0.7977 for NAA/Cr. When including dynamic FET information, 95% sensitivity and 100% specificity could be reached (AUC 0.970, *p* = 0.001). The coefficients were 33.76 for NAA/Cr, 4.36 for Cho/Cr, 98.35 for TBRmean, and 0.446 for TTP.

### 3.2. Differentiation between Glioblastoma and Non-Glioblastoma

**Figure 3:**
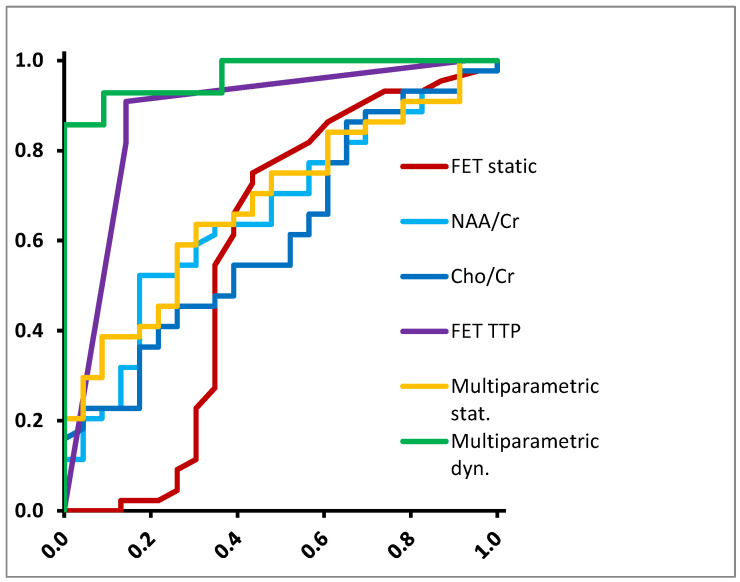
ROC curves for differentiation between glioblastoma and non-glioblastoma.

Second, we repeated the test for the differentiation between glioblastoma and non-glioblastoma (Table 3, Figure 3). In this case, dynamic FET TTP and NAA/Cr ratio were the only significant single parameters with sensitivities and specificities of 82% and 71% (AUC 0.792, *p* = 0.014, threshold 0.38) and 64% and 65% (AUC 0.662, *p* = 0.031, threshold 1.90), respectively. Using a multiparametric approach including dynamic FET-PET and MRS, accuracy could be improved substantially and reached a sensitivity of 93% and a specificity of 91% (AUC 0.968, *p* < 0.001). The model coefficients β were −1.554 for NAA/Cr, 1.136 for Cho/Cr, 0.001 for TBRmean and −0.328 for TTP, meaning that early TTP values correlated with higher tumor grade as expected.

### 3.3. Survival Analysis

**Figure 4:**
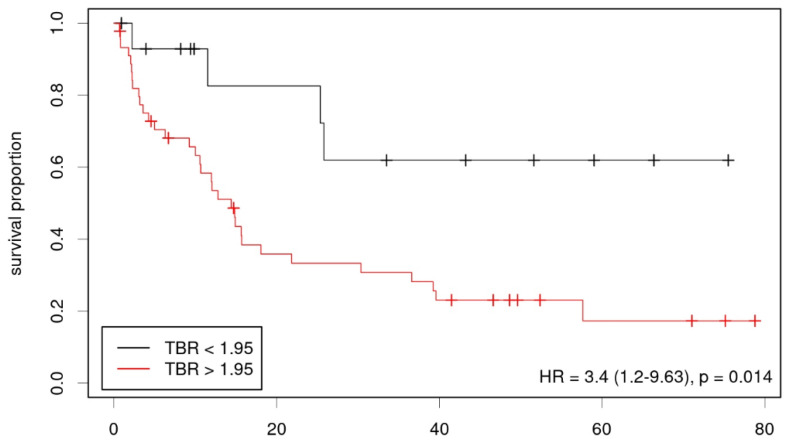
Kaplan–Meier plot showing low- and high-risk groups according to static FET-PET (TBR).

**Figure 5:**
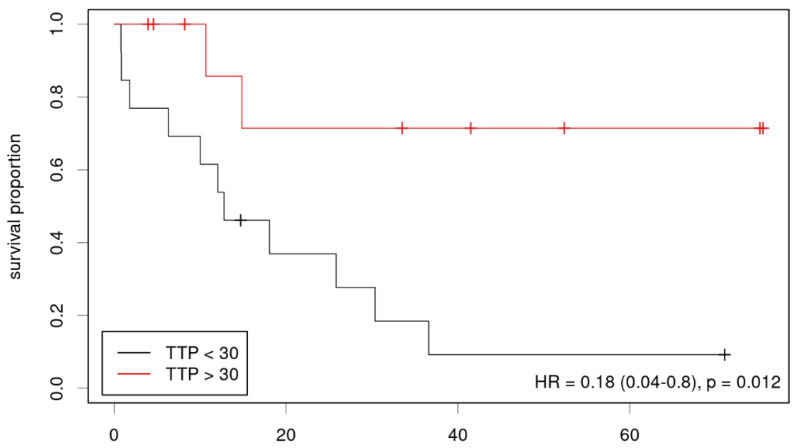
Kaplan–Meier plot showing low- and high-risk groups according to dynamic FET-PET (TTP).

**Figure 6:**
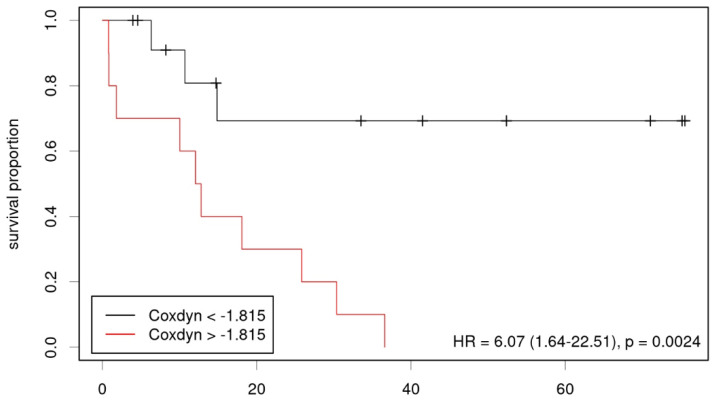
Kaplan–Meier plot showing low- and high-risk groups according to multiparametric combination.

Lastly, a possible correlation between OS and imaging parameters was determined (Table 4). Using the Kaplan–Meier analysis, we found static FET uptake and dynamic FET TTP, but not the MRS parameters significantly correlated with survival (HR = 3.4, *p* = 0.014, threshold TBR 1.95, Figure 4 and HR = 0.18, *p* = 0.012, threshold 30 min, Figure 5). In multivariate analysis using Cox regression, only TTP was confirmed as a significant predictor of survival, however in the considerably smaller cohort who had undergone dynamic imaging. A multiparametric approach using results from the Cox regression showed an improved prediction of overall survival if PET, as well as MRS-based parameters were taken into account (HR 6.07; *p* = 0.002, parameters (β) for Cox regression: 0.148 for FET TBR, −0.096 for FET TTP, −0.014 for Cho/Cr, −0.036 for NAA/Cr, Figure 6).

## 4. Discussion

Although multiparametric imaging can be called a new radiologic paradigm, studies quantitatively analyzing its value in gliomas are few. This fact is rather surprising, considering the substantial difficulties conventional MRI is facing in this entity. Determination of tumor subtype and grading, for example, is challenging though desirable for therapy planning, particularly in locations where biopsies are difficult to acquire. Several advanced MR techniques such as diffusion- and perfusion-weighted imaging and MR spectroscopy, as well as PET with glucose and amino acid tracers, have been investigated for this purpose, but sensitivities and specificities remain far from optimal. Consequently, in many cases, follow-up imaging remains the only way to assess the malignancy of a lesion.

Multimodal imaging promises to combine the advantages and compensate for the downsides of the single imaging modalities and therefore enhance the overall diagnostic accuracy [23]. The combination of PET and MRS is of particular interest, given the high potential of both techniques to elucidate molecular processes in brain tumors. A review of the existing studies of relevance can be found in [16]. In this work, we focused on the multiparametric assessment of glioma grade and prognosis using MRS and FET-PET, a topic that has not yet been studied in detail.

In the first step, we established the diagnostic value of static and dynamic FET-PET and MRS in our cohort of patients with intracranial gliomas. Both modalities were able to discriminate between low-grade and high-grade tumors according to the ROC analysis, which is in line with single-modality studies [19,24], but in contrast to an earlier publication on multimodal imaging only showing significant results for FET, but not for spectroscopy [18]. However, in our study, we included a substantially higher number of patients (67 vs. 38) and performed multi-voxel MRS, which could better depict the intratumoral heterogeneity, a hallmark of glial cell tumors [25,26]. That said, FET-PET still outperformed MRS for this purpose (AUC 0.86 for PET TBR vs. 0.72 for Cho/Cr ratio). Differentiation between glioblastoma and non-glioblastoma was likewise possible with PET and MRS, although the sensitivity and specificities of parameters differed: dynamic FET imaging offered increased diagnostic value compared to static PET, and NAA/Cr outperformed Cho/Cr. 

The multimodal analysis showed substantial benefit, both for the differentiation between low and high-grade gliomas and the detection of glioblastomas. With a combination of PET and MRS parameters, the diagnostic accuracy could be increased to an AUC of 0.97 compared to 0.86 and 0.88 for the best single modality. In the first case, it was sufficient to combine only static FET-PET and NAA/Cr ratios, while in the second case, static and dynamic PET as well as MRS parameters were included to obtain a multiparametric discriminator.

Prognostication is another important issue, especially for lesions in deeper locations or in eloquent areas which are difficult to biopsy, resect, and/or irradiate. Decisions on whether to stick to a wait-and-see strategy or to escalate therapy depend largely on the prognosis of the respective patient. In our study, static and dynamic FET-PET were both correlated with overall survival, which is in accordance with earlier studies [11,27], with dynamic TTP being the only significant covariate in multivariate analysis. The MRS parameters showed no clear correlation with prognosis; accordingly, we could not find clear evidence of a correlation between Cho/Cr and NAA/Cr ratios with overall survival in the literature. Instead, most authors relied on a more sophisticated spectroscopic technique to improve prognostication in human gliomas [28]. However, the inclusion of both FET-PET and MRS in a multiparametric approach was again beneficial when estimating patient survival. One possible reason for the relatively low predictive value of standard MRS could be the high intratumoral heterogeneity of gliomas. With higher resolution multi-voxel techniques, it should be easier to identify hot spots of higher malignancy within the tumor. We looked at maximum values for FET uptake and metabolite ratios and our results for MRS were better than those who used single voxel spectroscopy [18]. That said, newer studies use even lower voxel sizes [29], possibly reducing partial volume/averaging effects and further improving the diagnostic accuracy of MRS.

Our results are of particular interest in the light of growing use of hybrid PET/MRI scanners for brain imaging. The combined imaging protocol offers increased convenience for the patient, within parallel time-saving PET and MR imaging and excellent temporal and spatial co-registration [30,31]; however, scanning time is still restricted and sequences have to be selected carefully. As our work suggests, MRS promises to be a reasonable adjunct to amino acid PET, especially for the initial work-up of intracranial gliomas. In this respect, PET-guided MRS is a sophisticated technique, which aims to focus MRS on a hot spot determined by FET-PET [32].

Limitations of our study include the still relatively low number of patients, especially with dynamic FET-PET and low-grade gliomas, which reduces the validity particularly for the discrimination of low-grade gliomas by dynamic imaging; the retrospective study design, and, consequently, the lack of a consistent molecular pathologic evaluation e.g., with respect to IDH, MGMT, and 1p19q status, all of which could be addressed in future prospective studies, preferably performed in a hybrid PET/MRI setting.

## 5. Conclusions

FET-PET and MRS provide complementary information, which can be used to better characterize gliomas before therapy. These results are particularly interesting with respect to hybrid PET/MRI, which can acquire both modalities in one imaging session.

## Figures and Tables

**Table 1:** Patient demographics.

Patients, N	67
Mean age, y	55.0 ± 16.3
Sex, n (%)	
Female	29 (43.3)
Male	71 (56.7)
Recurrent tumor, n (%)	
no	60 (89.6)
yes	7 (10.4)
Histology, n (%)	
Diffuse astrocytoma WHO °II	5 (7.5)
Oligodendroglioma WHO °II	4 (6.0)
Anaplastic astrocytoma WHO °III	10 (14.9)
Anaplastic oligodendroglioma WHO °III	4 (6.0)
Glioblastoma WHO °IV	44 (65.7)
Median OS, m (range)	12 (1–78)
Resection status, n (%)	
Gross total	30 (44.8)
Partial	29 (43.3)
Biopsy	8 (11.9)

**Table 2:** Diagnostic accuracy of MRS and PET parameters for differentiation between low and high-grade gliomas. Significant results are marked with an asterisk.

	AUC	Cut-Off	*p*	Sensit.	Specif.
FET TBR	0.855 (0.713–0.998)	2.00	0.001 *	86.2%	77.8%
FET TTP	0.790 (0.613–0.967)	20 min	0.049 *	100%	65.0 %
NAA/Cr	0.678 (0.514–0.843)	1.89	0.087	65.5%	55.6%
Cho/Cr	0.716 (0.497–0.934	2.22	0.039 *	86.2%	66.7%
Multipar. (stat.)	0.898 (0.777–1.000)	--	<0.001 *	93.1%	88.9%
Multipar. (dyn.)	0.970 (0.905–1.000)	--	0.001	95.0%	100%

**Table 3:** Diagnostic accuracy of MRS and PET parameters for differentiation between glioblastoma and non-glioblastoma. Significant results are marked with an asterisk.

	AUC	Cut-Off	*p*	Sensit.	Specif.
FET TBR	0.579 (0.498–0.749)	2.15	0.294	75.0%	66.5%
FET TTP	0.792 (0.606–0.979)	0.38	0.014 *	81.8%	71.4%
NAA/Cr	0.662 (0.527–0.796)	1.90	0.031 *	63.6%	65.2%
Cho/Cr	0.612 (0.472–0.752)	2.60	0.134	54.5%	64.0%
Multipar.	0.955 (0.880–1.000)	--	<0.001 *	100.0%	81.8%

**Table 4:** Univariate (Kaplan–Meier) and multivariate (Cox regression) correlation between OS and imaging parameters. Significant results are marked with an asterisk.

	Univariate		Multivariate	
	HR	*p*	HR	*p*
static FET TBR	3.40 (1.20–9.63)	0.014 *	1.25 (0.72–2.16)	0.428
dynamic FET TTP	0.18 (0.04–0.80)	0.012 *	0.90 (0.82–0.98)	0.011 *
NAA/Cr	1.49 (0.65–3.42)	0.340	0.91 (0.50–1.66)	0.768
Cho/Cr	2.26 (0.88–5.80)	0.082	0.98 (0.86–1.13)	0.821
Multipar.	6.07 (1.64–22.51)	0.002 *		

## Data Availability

All data are available upon reasonable request from the corresponding author. The data are not publicly available due to protection of the privacy of patients.
